# Quantification of the endogenous growth hormone and prolactin lowering effects of a somatostatin-dopamine chimera using population PK/PD modeling

**DOI:** 10.1007/s10928-020-09683-3

**Published:** 2020-04-04

**Authors:** Michiel J. van Esdonk, Jacobus Burggraaf, Marion Dehez, Piet H. van der Graaf, Jasper Stevens

**Affiliations:** 1 Division of Systems Biomedicine and Pharmacology, Leiden Academic Centre for Drug Research, Leiden University, Leiden, The Netherlands; 2 Centre for Human Drug Research, Leiden, The Netherlands; 3 Ipsen Innovation, Les Ulis, France; 4 Certara QSP, Canterbury, UK; 5 Department of Clinical Pharmacy and Pharmacology, University of Groningen, University Medical Center Groningen, Groningen, Netherlands

**Keywords:** Growth hormone, Deconvolution, Prolactin, Population PKPD, Dopastatin

## Abstract

A phase 1 clinical trial in healthy male volunteers was conducted with a somatostatin-dopamine chimera (BIM23B065), from which information could be obtained on the concentration-effect relationship of the inhibition of pulsatile endogenous growth hormone and prolactin secretion. Endogenous growth hormone profiles were analyzed using a two-step deconvolution-analysis-informed population pharmacodynamic modeling approach, which was developed for the analyses of pulsatile profiles. Prolactin concentrations were modelled using a population pool model with a circadian component on the prolactin release. During treatment with BIM23B065, growth hormone secretion was significantly reduced (maximal effect [E_MAX_] = − 64.8%) with significant reductions in the pulse frequency in two out of three multiple ascending dose cohorts. A circadian component in prolactin secretion was identified, modelled using a combination of two cosine functions with 24 h and 12 h periods. Dosing of BIM23B065 strongly inhibited (E_MAX_ = − 91%) the prolactin release and demonstrated further reduction of prolactin secretion after multiple days of dosing. This study quantified the concentration-effect relationship of BIM23B065 on the release of two pituitary hormones, providing proof of pharmacology of the chimeric actions of BIM23B065.

**Electronic supplementary material:**

The online version of this article (10.1007/s10928-020-09683-3) contains supplementary material, which is available to authorized users.

## Introduction

The pituitary is a key endocrine gland that produces a wide variety of hormones, including growth hormone (GH) and prolactin (PRL) [1]. In acromegaly, a pituitary adenoma causes disruption in the highly regulated mechanisms that control the stimulation and inhibition of GH [2]. Pituitary adenomas cause severe GH hypersecretion [3, 4] and may also lead to an excessive release of PRL in 20–30% of patients [2, 4].

Recently, a placebo-controlled single ascending and multiple ascending dose phase I clinical trial was performed to study the pharmacokinetics (PK), safety and tolerability of BIM23B065 in healthy male volunteers [5]. BIM230B065 belongs to the novel class of dopastatins, which concurrently target somatostatin and dopamine receptors and is under investigation for the treatment of neuro-endocrine tumors [6]. The effects of BIM23B065 on endogenous GH and PRL secretion has previously been reported on a per-cohort basis. However, no concentration-effect relationship between BIM23B065 and GH and PRL secretion has yet been established.

To quantify the pharmacokinetic/pharmacodynamic (PK/PD) relationship on endogenous GH secretion, commonly the mean of multiple GH observations [7, 8], the area under the GH-concentration–time curve [9], or a simplification of the circadian rhythm of GH was used [10]. However, these methods do not incorporate the high intra- and inter-individual variability in pulsatility that is characteristic of endogenous GH profiles. Therefore, these are at best an empirical way to quantify a drug effect and have limited utility for the prediction of drug effects with new dosing regimens. For PRL response modelling, different structural PD models with different levels of complexity (turnover model, pool model, agonist–antagonist interaction and combinations of a pool model with a feedback loop [11–14]) are reported in the literature with some including circadian rhythmicity modelled as two cosine functions with 12 h and 24 h periods [11, 13].

The aim of this study was to quantify the PK/PD relationship between BIM23B065 plasma concentrations and the endogenous GH secretion, while taking into account an individual’s pulsatile profile, and of the PRL secretion, using non-linear mixed effects (NLME) modelling in healthy male volunteers.

## Methods

### Study design

A phase 1, double-blind, randomized, placebo-controlled single (S.A.D.) and a 13-day multiple ascending dose (M.A.D.) clinical trial was performed to primarily investigate the PK, safety, and tolerability of subcutaneously administered BIM23B065 [5]. In short, a total of 64 healthy male volunteers were included in the study, of which one individual withdrew from the study before dose administration and was not replaced. Cohorts consisted of 8 individuals (active n = 6, placebo n = 2 per cohort) and received doses of 0.1 mg, 0.4 mg, 0.8 mg, 1.2 mg or 1.5 mg in the S.A.D. cohorts or 1.2 mg once daily (q.d.), 0.8 mg twice daily (b.i.d.) and 1.0 mg b.i.d. in the M.A.D. cohorts, administered at 8 h/16 h intervals.

Endogenous GH and PRL sampling was performed once during the S.A.D. and twice, on day 7 and day 12, during the M.A.D. part of the study. Sampling started 2 h before dose administration in the S.A.D. part and 1 h before dosing in the M.A.D part of the study. Sampling was performed up until 12 h after dosing in the S.A.D part and 11 h after dosing in the M.A.D. part at 20 min intervals, except in the first hour after dosing of the S.A.D. cohorts, where it followed the PK sampling schedule (15, 30, 60 min after dosing). The GH samples were analyzed using the Immulite 2000 assay (WHO IS 98/572) and the PRL samples using a two-step immunoassay (Architect Prolactin assay).

### GH model development

All individual GH profiles were analyzed with a deconvolution analysis in which the baseline secretion, the elimination rate, and the pulsatile secretion events were extracted from an individual profile. The pulsatile secretion events were assumed to follow a Gaussian shape and the optimal number, the location of secretion events, and the Gaussian pulse width of an individual were determined. This methodology has been shown to have high sensitivity and specificity in the identification of pulses in endogenous pulsatile hormonal profiles [15].

The individual deconvolution analysis of GH profiles was performed in AutoDecon (developed by Johnson et al*.*) which requires regularly spaced observations, an initial pulse secretion, and an initial half-life as input [15]. Therefore, a data transformation was performed to maintain the required regularly spaced 20 min sampling interval. As such, the time points at 15 and 30 min after dosing in the S.A.D. cohorts were shifted by 5 and 10 min respectively to maintain a 20-min interval throughout the full observation period. The initial pulse secretion width was set to half of the sampling interval (10 min) and the initial GH half-life was set to 15 min. The pulse frequency, obtained from the deconvolution analysis, of BIM23B065 treated individuals was analyzed for significance (generalized linear model with Poisson distribution, p < 0.05) compared with the placebo cohort. The pulse frequency and the location of pulses from the deconvolution analysis were converted to a format suitable for population NLME modelling in NONMEM [16, 17].

The deconvolution-informed PD modeling of endogenous GH profiles followed a sequential modelling procedure. First, modelling started with the estimation of the population parameters in placebo treated individuals. Inter-individual variability (IIV) in the population parameters and between-occasion variability (BOV) between day 7 and day 12 was included following a bottom-up inclusion procedure. Then, the estimated population parameters and variance distributions were fixed to the placebo estimates and model development continued with the full dataset containing both placebo and BIM23B065 treated individuals [17]. Multiple PK/PD relationships, linear and (sigmoidal) maximal effect (E_MAX_), driven by the plasma PK of BIM23B065 or via an effect compartment, were tested for significance on the baseline secretion and pulse amplitude parameters during model development [18]. The E_MAX_ relationship in which the hill coefficient (*γ)* was estimated or fixed to 1, and where the E_MAX_ parameter was estimated or fixed to − 100% on the pulse amplitude (assuming a full inhibition of GH secretion) was explored.

### PRL model development

The data from this study did not include any information on the concentrations of inhibitory feedback hormones for PRL. Furthermore, no dose administrations with short consecutive dosing intervals were administered, which complicated the estimation of feedback mechanisms on PRL secretion and therefore model development focused on the quantification of a pool model, as proposed by Movin-Osswald et al. [14]. As a circadian component in the release of PRL secretion was expected [19], all time points in the dataset were normalized to 6 a.m. and the model included a 24 h initialization period. The periodicity of the circadian rhythm of PRL secretion was explored by implementing a cosine function (Eq. 1) in the structural model while only including data from the placebo treated individuals.

1 $$\mathrm{D}\mathrm{I}\mathrm{U} = {\Theta }_{Amplitude}\cdot \mathrm{cos}(2\pi \cdot \frac{t-{\Theta }_{Phaseshift}}{period})$$

where DIU is the diurnal effect over time (*t*), *Θ*_*Amplitude*_ is the height of the peak (or trough) of the cosine function from the mesor, *Θ*_*Phaseshift*_ is the horizontal shift in the cosine and *period* is the time needed to complete a single cycle. Multiple periods, and combinations of 2 cosine functions, were investigated to account for the circadian rhythm observed in the data. Periods were chosen so that all cycles were completed in a 24 h period. The circadian rhythm was implemented on the PRL release rate constant (*k*_*r*_) from the pool compartment. The PRL-inhibiting effect of BIM23B065 was investigated by the use of linear or sigmoidal E_MAX_ PK/PD relationships on the *k*_*r*_.

### Inter-individual variability, between-occasion variability and covariates

In both models, the IIV and BOV were included following a bottom-up inclusion procedure. Both IIV (*η)* and BOV (*ϰ)* were assumed to follow a log-normal distribution (except phase shift parameters) and were included when a significant improvement (p < 0.01) in model fit was observed and the numerical stability of the model was improved. The covariates age, weight, body mass index, height and lean body mass were investigated as descriptors of the identified IIV. Correlations between the post hoc Bayesian estimates and the covariates were evaluated and tested for inclusion in the structural model when a correlation (r^2^ > 0.50) was present. Covariates were centered around their mean values upon inclusion in the model. Covariates were included using a forward inclusion method (p < 0.05) followed by backward deletion (p < 0.01).

### Model evaluation

Model selection and evaluation was based on a significant (p < 0.01) drop in the objective function value (OFV) of 6.64 points between nested models after the addition of one degree of freedom, goodness of fit (GOF) plots and additional numerical evaluation with a focus on the relative standard errors (RSE) of population parameters and the η-shrinkage. In general, RSEs of population parameters were considered acceptable when below 50% and η-shrinkage should not exceed 30% [20]. When the covariance step in NONMEM was not completed, RSEs were computed from successfully minimized models in a non-parametric bootstrap of 50 samples.

Models were visually judged for bias on the basis of GOF figures, which included the individual (IPRED) and population (PRED) model predictions versus observations and conditional weighted residuals with interaction (CWRESI) versus PRED and time after dose [20]. The IPRED and PRED versus observations should show a scatter around the line of unity whereas the CWRESI over PRED and time after dose should show a homogenous scatter around 0 with the majority of data points between the [− 2, 2] interval. When cosine functions were included in a model to account for circadian variability, CWRESI over time of day was explored to identify a circadian bias. When computational power constraints and model run times were acceptable (< 2 days), a non-parametric bootstrap analysis was performed, using 1000 samples, as interval validation for the calculation of the median and the 95% confidence intervals of the parameter estimates. For the PRL model, a prediction corrected visual predictive check (pcVPC) was created over clock time. No pcVPC could be created for the GH model as model diagnostic due to the differences in the timing of pulses. Therefore, normalized prediction distribution errors (NPDE) were computed for all models, stratified per treatment day, to evaluate model predictions.

### Software

Data transformation and graphical analysis was performed in R (V3.4.0) [21]. Deconvolution analysis was performed using AutoDecon (V20090124) [15]. NLME modeling was performed in NONMEM V7.3 [16] in conjunction with Perl-speaks-NONMEM V4.6.0 [22]. All models were estimated with the first-order conditional estimation with interaction (FOCEI) method applying user-written ordinary differential equations in ADVAN 9 or 13. The non-parametric bootstrap was performed using the ‘bootstrap’ command in Perl-speaks-NONMEM.

## Results

A population PK analysis on BIM23B065 has previously been performed [23]. In short, the PK of BIM23B065 was best described by a 2-compartment model with both linear and non-linear elimination kinetics. The individual post-hoc Bayesian estimates of the previously published PK model were used to simulate the individual concentration–time profiles of BIM23B065 for the PK/PD analysis.

### GH model development

**Fig. 1:**
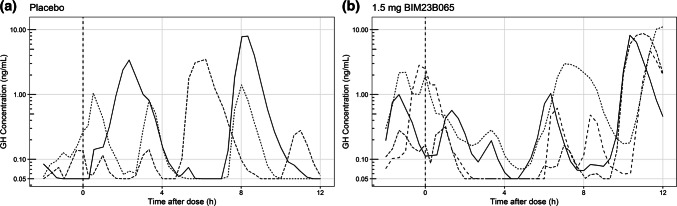
Individual concentration–time growth hormone profiles of representative placebo treated subjects (**a**) and BIM23B065 treated subjects in the 1.5 mg cohort (**b**). Dashed vertical line at 0 h represents time of dose administration. Data below the lower limit of quantification was fixed to 0.05 ng/mL

For endogenous GH profiles, several individuals did not have any observations above the lower limit of quantification (LLOQ) of 0.05 ng/mL during the observation period (1 placebo, 3 BIM23B065 treated). These individuals were therefore excluded from further analysis. A total of 77 12 h profiles from 59 individuals consisting of 3054 GH observations (776 from placebo and 2278 from BIM23B065 treated individuals) were used for model development. Within the placebo group, 20% of the observations were below the LLOQ. Within the treated group, this percentage increased to 43%. The observations below the LLOQ were fixed to the LLOQ of 0.05 ng/mL in this analysis to maintain the estimation of basal GH secretion. Figure 1 shows the concentration–time profiles of multiple representative placebo- and 1.5 mg BIM23B065-treated individuals, visualizing the high level of variability between individuals and between the pulses within an individual.

The median pulse interval, estimated in the deconvolution analysis, in the placebo cohort was 74 min (IQR 25%-75% = 44–160 min, 95% upper boundary = 293 min). The pulse frequency showed a small but significant reduction in the 1.2 mg q.d. (p = 0.013) and the 0.8 mg b.i.d. (p = 0.05) cohorts compared with placebo (Online Resource 1). In general, a wide range of pulse frequencies was estimated by the deconvolution analysis in the individual GH profiles after either placebo or BIM23B065 treatment.

Due to the high number of observations below the LLOQ, the large interval between two pulses could be the result of GH inhibition by BIM23B065. However, since these inhibited pulses cannot be identified in a deconvolution analysis, this would result in missing information on the concentration-effect curve. Hence, no observations at the maximum effect (a fully inhibited pulse) are available. If the pulse interval between two identified pulses was higher than 300 min, a > 95% probability of an unidentified pulse was expected. Therefore, an additional pulse location in the middle of these two identified pulses was added to account for this. This was done for both placebo and treated individuals to prevent a selection bias. As a result, a total of 34 new pulse locations were included (4 placebo-, 30 BIM23B065-treated pulses) in the dataset.

In the placebo model, significant IIV was estimated on the elimination rate constant (ΔOFV = − 264.8), the pulse secretion width (ΔOFV = − 162.4), and the GH baseline secretion (ΔOFV = − 125.1). The inclusion of BOV was significant on the elimination rate constant (ΔOFV = − 102.4, coefficient of variation [CV] = 8.8%) and the pulse secretion width (ΔOFV = − 43.4, CV = 33.2%). A proportional residual error structure was superior over an additive or a combined residual error structure.

**Fig. 2:**
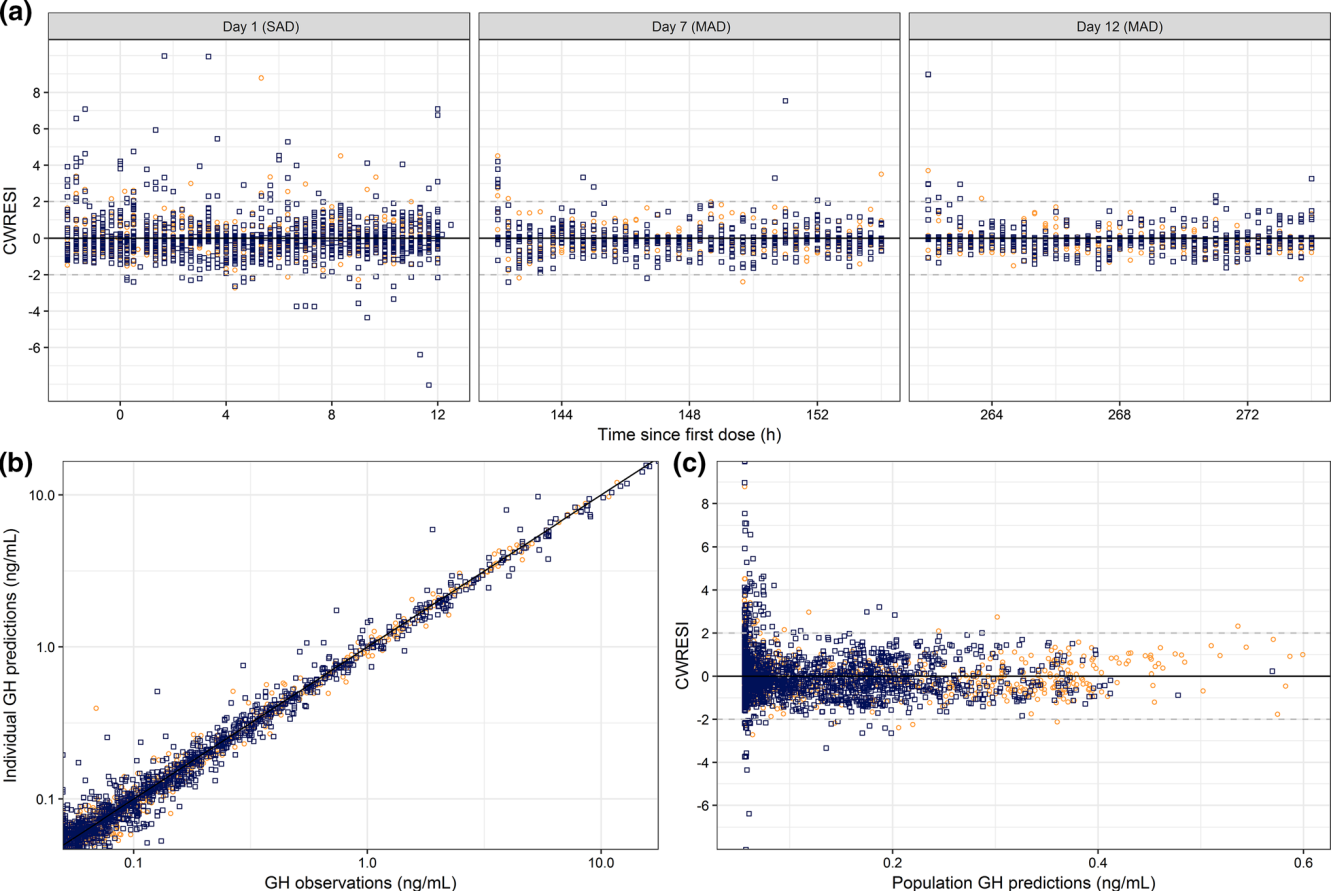
Goodness of fit plots of the developed endogenous growth hormone model. **a** Conditional weighted residual with interaction (CWRESI) versus time since first dose for day 1, 7 and 12. **b** Individual model predictions versus GH observations. **c** CWRESI versus population growth hormone predictions. Orange dots = placebo observations, blue squares = BIM23B065 treated observations, black diagonal line = line of unity, grey dashed line = [− 2, 2] interval (Color figure online)

Placebo parameters were estimated with low RSEs (< 10%) with high levels of inter-pulse variability in the pulse amplitude (CV = 555%). No structural bias could be identified in the CWRESI versus time which showed the majority of the points between the [− 2, 2] interval, the population model predictions showed a broad scatter at the lowest regions, indicating a wide distribution in the model fit at the baseline levels (Fig. 2). The individual model predictions were close to the line of unity, indicating an adequate model fit for placebo-treated individuals.

After including the data from BIM23B065-treated individuals, the inclusion of an E_MAX_ concentration-effect relationship of BIM23B065 on the pulse secretion, originating from an effect compartment, gave the largest drop in OFV (ΔOFV = − 55.4) relative to an absence of drug effect and was superior to a linear effect (ΔOFV compared to no effect = − 38.1). The E_MAX_ was estimated as a 64.8% inhibition of the secretion of GH with an EC_50_ of 0.609 µg/L. The estimation of a hill coefficient did not improve the model fit and was therefore fixed to 1. The inclusion of an additional effect that reduced the basal secretion of GH was not superior to the parent model. A drug effect on the basal secretion of GH might be identified when a more sensitive analysis assay is applied.

The inclusion of IIV on the EC_50_ resulted in a small, but significant, decrease in the OFV of 8 points, but with a very high variance (ω^2^ = 12.1). This indicates the existence of high variability in the EC_50_ within this population with only limited improvement in the individual model fit. Furthermore, a decrease in numerical stability was observed in this model after inclusion of the variance on the EC_50_, and was therefore excluded from the model. No significant covariates were identified for inclusion.

**Fig. 3:**
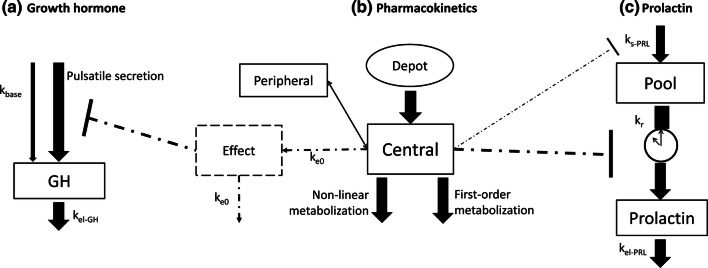
Structural population models for the endogenous GH secretion (**a**), the pharmacokinetics of BIM23B065 (**b**) and the endogenous prolactin secretion (**c**). Inhibitory dotted lines indicate inhibition via E_MAX_ equations. Clock indicates parameter with circadian rhythm. k_base_ = baseline secretion, k_el-x_ = elimination rate constant, k_e0_ = effect compartment rate, k_s-PRL_ = prolactin synthesis rate, k_r_ = release rate

The estimated model parameters for the system specific parameters of GH and the concentration–effect relationship of BIM23B065 on endogenous GH secretion are shown in Table 1. The GOF plots of the developed model for placebo- and active-treated individuals are depicted in Fig. 2. The individual model predictions are scattered close to the line of unity. A larger distribution of the CWRESI at the lowest population predictions was observed, with no bias in the CWRESI over time since first dose during the three observation days, with the majority of the model predictions within the [− 2, 2] interval. The condition number was moderate with a value of 54.4. The structural model for endogenous GH secretion is depicted in Fig. 3a. Due to computational power restrictions, no non-parametric bootstrap was performed. The NPDE results are depicted in Online Resource 2, showing a normal distribution of the observations in the Q-Q plot, with a small underestimation of the median.

**Table 1:** Model parameter estimates for the population pharmacodynamic model of the endogenous growth hormone secretion

Parameter	Units	Estimate [RSE%] (CV%)
Structural model parameters^a^		
Baseline	ng/mL	0.056 [0.4]
Secretion width	h	0.184 [1.16]
Amplitude	ng/mL	1.69 [1.6]
k_el-GH_	/h	3.6 [1.38]
Drug effect parameters		
Effect compartment rate	/h	1.25 [4.97]
E_MAX_	%	− 64.8 [2.7]
EC_50_	µg/L	0.609 [41.7]
γ	–	1^b^
Inter-individual variability		
ω^2^ baseline	–	0.0288 (17.1)
ω^2^ secretion width	–	0.0434 (21)
ω^2^ k_el-GH_	–	0.225 (50.2)
ω^2^ amplitude_n_	–	3.46 (555)
ω^2^ BOV secretion width	–	0.104 (33.2)
ω^2^ BOV k_el-GH_	–	0.00775 (8.82)
Residual error structure		
σ^2^ proportional error	–	0.0247

*RSE* relative standard error, *CV%* coefficient of variation, *95% CI* 95% confidence interval, *Amplitude*_*n*_ variability between n pulses within an individual

^a^Structural model parameters were estimated on placebo data only

^b^Indicate fixed parameter

### PRL model development

A total of 3116 PRL observations were available for model development, of which 796 were PRL concentrations of placebo treated individuals, used for the structural model development with circadian rhythm, and 2320 PRL concentrations were available from BIM23B065-treated individuals. No samples were below the LLOQ.

Visual inspection of the placebo data indicated a circadian rhythm in the release of PRL, with the time period during which concentrations were low (bathyphase) in the morning and higher PRL concentrations during the afternoon (Online Resource 3). This was best described using a combination of two cosine functions with 24 h and 12 h periods on the *k*_*r*_ from the pool compartment, giving a 266 point reduction in the OFV compared to a steady state release, and was superior over other cosine period combinations.

The inclusion of an inhibitory E_MAX_ drug effect on the *k*_*r*_, driven by the PK of BIM23B065 gave a 1518 point reduction in the OFV. An E_MAX_ concentration-effect relationship was superior over a linear effect (ΔOFV = − 938 compared to no drug effect) and over a sigmoidal E_MAX_ relationship of which the hill factor could not be accurately estimated.

Graphical model evaluation suggested an overprediction for individuals in the b.i.d. cohorts of the M.A.D. part of the study, indicating that the typical PRL concentrations were reduced after 7 or 12 days of treatment with BIM23B065 (Online Resource 4A). To investigate whether prolonged dosing of BIM23B065 would decrease the synthesis of PRL in the pool compartment (*k*_*s-PRL*_), it was investigated if, and to what extent, significant differences between day 1 (S.A.D.), day 7 and day 12 (M.A.D.) existed, driven by the cumulative exposure (mg*h/L) over time to BIM23B065 using Eq. 2.

2 $$I(t) = \frac{BIM23B065 exposure\left(t\right)\cdot {\Theta }_{Slope}}{1+BIM23B065 exposure\left(t\right)\cdot {\Theta }_{Slope}}$$

In which the *Θ*_*Slope*_ parameter determines the steepness of the inhibition curve and the *I*(*t*) remains between 0 (no inhibition) and 1 (full inhibition). The inclusion of a decrease in *k*_*s-PRL*_ over time, driven by the exposure to BIM23B065, resulted in a 66 point reduction in the OFV, with a *Θ*_*Slope*_ of 2.73. This improved the population model fit to a more homogenous scatter around the line of unity (Online Resource 4B).

Significant IIV was identified on the, in order of inclusion, *k*_*s-PRL*_ (ΔOFV = − 2009), and on the *Θ*_*Amplitude*_ of the 12 h (ΔOFV = − 365) and 24 h (ΔOFV = − 298) cosine functions. A proportional residual error structure was best fit for purpose. No significant covariates were identified.

**Fig. 4:**
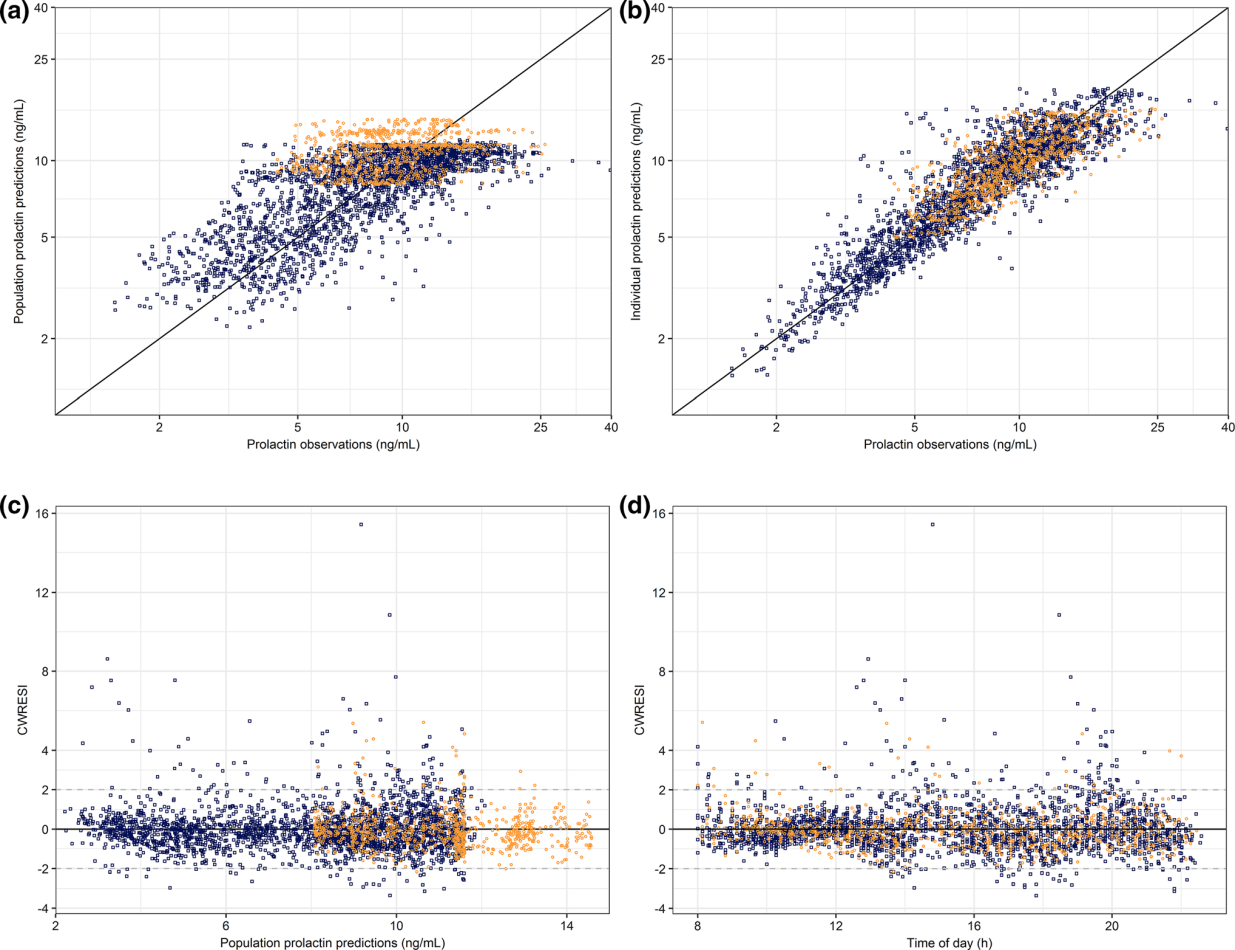
Goodness of fit plots of the developed endogenous prolactin model. **a** Population model predictions versus observations, **b** individual model predictions versus observations, **c** conditional weighted residuals with interaction (CWRESI) versus population predictions, and **d** CWRESI versus time of day. Orange dots = placebo observations, blue squares = BIM23B065 treated observations, black diagonal line = line of unity, grey dashed line = [− 2, 2] interval (Color figure online)

The estimated model parameters for the developed PRL model are shown in Table 2. The structural model for endogenous PRL secretion is depicted in Fig. 3c. The GOF plots of the developed model for placebo- and BIM23B065-treated individuals are depicted in Fig. 4. A clear difference between the placebo and treated individuals can be observed, in which the PRL concentrations of placebo individuals are consistently higher. A scatter around the line of unity for the population and individual model predictions indicates adequate model fit with large variability in the population. The condition number was low with a value of 15.7.

**Table 2:** Model parameter estimates for the population pharmacodynamic model of the prolactin secretion

Parameter	Units	Estimate [RSE%] (CV%)	Shrinkage (%)	Bootstrap median (95% CI)
Population parameters				
k_r_	/h	0.011 [30.2]	–	0.011 (0.0009 to 0.260)
k_el-PRL_	/h	1.25 [9.55]	–	1.28 (1.03 to 1.63)
k_s-PRL_	ng/mL/h	13.3 [8.54]	–	13.6 (10.2 to 17.6)
Amplitude cos 24 h	–	0.168 [13.9]	–	0.177 (0.08 to 0.28)
Phase shift cos 24 h	h	17.3 [3.51]	–	17.1 (14.58 to 19.61)
Amplitude cos 12 h	–	0.095 [10.8]	–	0.088 (0.036 to 0.140)
Phase shift cos 12 h	h	10.2 [5.15]	–	10.3 (7.50 to 11.44)
E_MAX_	%	-91 [5.53]	–	− 91 (− 78.5 to − 99.65)
EC_50_	µg/L	1.27 [14.3]	–	1.18 (0.77 to 1.85)
Slope	–	2.73 [26.9]	–	2.36 (0.475 to 10.67)
Inter-individual variability				
ω^2^ k_s-PRL_	–	0.068 (26.4)	< 0.01	0.066 (0.046 to 0.091)
ω^2^ amplitude 24 h	–	0.57 (87.3)	11.08	0.55 (0.19 to 1.98)
ω^2^ amplitude 12 h	–	0.87 (118)	16.96	1.06 (0.47 to 3.83)
Residual error structure				
σ^2^ proportional error	–	0.049	2.21	0.048 (0.039 to 0.060)

Bootstrap results: 99.4% successful minimizations, 68.0% successful covariance steps runs

*RSE* relative standard error, *CV%* coefficient of variation, *95% CI* 95% confidence interval

The CWRESI versus time of day shows a minimal bias around 0, indicating that there is still remaining variability present that could not be quantified in the current model solely with the use of two cosine functions. The collection of additional data may result in the identification of another cosine function with a shorter period to account for this. No bias in the CWRESI versus the population predictions was identified, with the majority of the predictions within the [− 2, 2] interval. The highest CWRESI of 15.4 was the result of a high PRL pulse (concentrations up to 39.7 ng/mL) occurring between the two doses in the M.A.D. part of the study, where a PRL concentration of 13.3 ng/mL was estimated. This was also the case, but to a lesser extent, with other model predictions that had high CWRESI values. All RSE’s and η-shrinkage were below their acceptance criteria of 50% and 30%, indicating precise estimation of these parameters. Bootstrap medians and confidence intervals were close to the estimated values. The NPDE and pcVPC results are depicted in Online resource 5, showing normal distributions of the distribution errors on all days and accurate description of the typical individual and the variability of the population over clock time. The NONMEM model codes for the GH and PRL models are available in Online Resource 6.

**Fig. 5:**
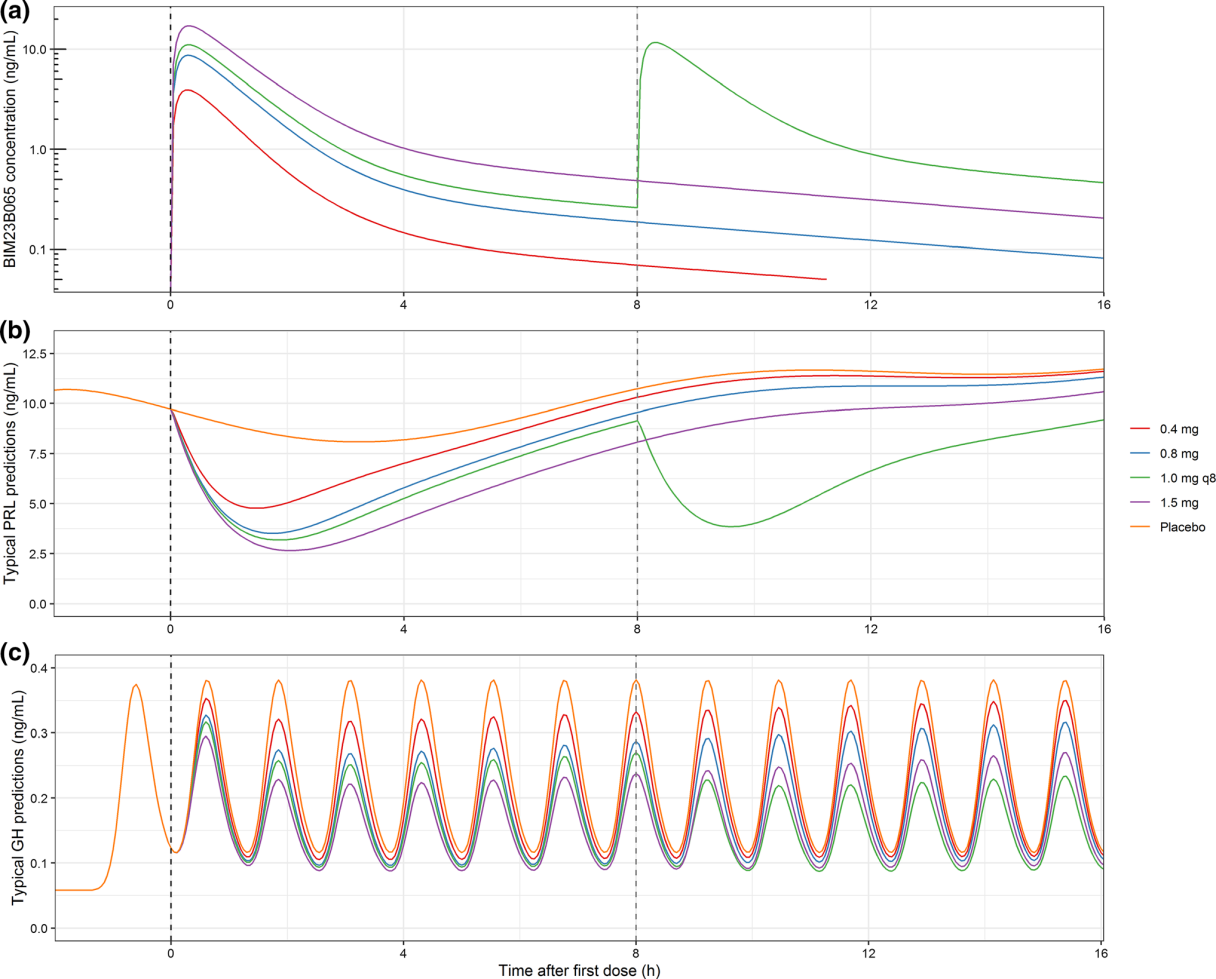
Simulations of a typical PK/PD response after receiving placebo, 0.4, 0.8, or 1.5 mg single dose at time 0 h, or 1.0 mg with 8 h intervals (q8). **a** pharmacokinetics of BIM23B065, **b** typical response profile on the PRL secretion, **c** typical response profile on the GH secretion. Prolactin (PRL) simulation was performed with dosing at 8 am and 4 pm. Growth hormone (GH) secretion was simulated with the typical pulse amplitude and pulse frequency, without variability between pulses

A simulated effect of BIM23B065 on a typical endogenous PRL and GH profile over time, at multiple dosing levels of BIM23B065, is depicted in Fig. 5. A large reduction in the secretion of both pituitary hormones can be observed compared with the typical placebo profile.

## Discussion

BIM23B065 was able to significantly inhibit the endogenous secretion of GH and PRL by 64.8% and 91%, respectively. The quantified GH lowering properties of BIM23B065 show similarities with the in vitro established inhibition of 63% in cultured human pituitary adenoma cells [24]. An effect compartment was included to account for a delay in the effect on the pulsatile GH profile. No strong reduction in GH pulse frequency was identified, with only small reductions observed in two out of three M.A.D. cohorts. For PRL, a strong and direct reduction was observed after both single and twice-daily BIM23B065 administrations, which lasted for approximately 8 h before returning back to baseline. The developed PK/PD models were able to accurately quantify the concentration-effect relationship after single and multiple doses of BIM23B065 on the endogenous secretion of GH and PRL in healthy male individuals.

In order to account for the underestimation of the ‘true’ number of pulses in the deconvolution analysis, a pulse location was added if an interval was larger than the 95%-percentile of the placebo data. The implementation of these pulses in the dataset informed the concentration-effect curve at the section of the maximal effect (informing on the parameter estimate of E_MAX_) and enabled the quantification of the inhibitory BIM23B065 effects with high accuracy in the parameter estimates. However, the high proportion of data below the LLOQ limited the precise estimation of the actual baseline GH secretion in the BIM23B065-treated individuals. The clear increase in the percentage of data below the LLOQ in the BIM23B065-treated versus placebo-treated individuals indicate that BIM23B065 may have had an effect on this baseline secretion, which could not be quantified in the current PK/PD model. Furthermore, the population baseline parameter may be overestimated and the variance on this baseline parameter may be underestimated by this approach, which should be taken into account in subsequent simulations. Due to the high level of variability, in future studies, a pre-treatment baseline day could be incorporated in the study design to better describe the endogenous hormonal secretion of an individual before administration of BIM23B065.

For PRL, the identified circadian rhythm on the release rate (two cosine functions with 24 h and 12 h periods) was similar to the pattern identified by others [13]. As there are no observations of feedback hormones in this study, which are applied in different PRL models in literature, a parsimonious structural model that was able to adequately describe the data, with high accuracy in the parameter estimates (RSE < 50%) was applied. A pool model to capture the endogenous PRL release in this study was best fit for purpose and quantified the significant inhibitory effects after both single- and multiple-doses of BIM23B065.

To the best of our knowledge, this is the first time that the concentration-effect relationship of a drug targeting endogenous pulsatile GH secretion has been analyzed while maintaining an individual’s pulsatile profile. This analysis method increased the amount of information that was obtained from a phase I clinical trial by the quantification of a concentration-effect relationship over time compared with a dose–response relationship based solely on summary statistics (e.g. mean, area under the curve). The identified concentration-effect relationships on both pituitary hormones (GH and PRL) provide information on the extent of the inhibitory effects of BIM23B065 and shows that BIM23B065 is able to reduce GH and PRL secretion in healthy male volunteers, indicative of both active somatostatin and dopamine moieties. Using the developed population models for GH and PRL, clinical trial simulations can be performed to identify the probability of success of new clinical trial study designs with BIM23B065. Additionally, the estimated parameters can be used as prior information in the study design of new compounds when investigating GH or PRL inhibition. Especially with the identified GH model, the effect of different simplified sampling protocols (e.g. 1 random GH sample, multiple GH sample every 10 min for 1 h, etc.) can now be simulated and can be taken into consideration in the design of new trials.

## Electronic supplementary material

Below is the link to the electronic supplementary material.

Supplementary file1 (DOCX 119 kb)

Supplementary file2 (DOCX 739 kb)

Supplementary file3 (DOCX 288 kb)

Supplementary file4 (DOCX 494 kb)

Supplementary file5 (DOCX 1084 kb)

Supplementary file6 (DOCX 44 kb)

## Data Availability

Where patient data can be anonymised, Ipsen will share all individual participant data that underlie the results reported in this article with qualified researchers who provide a valid research question. Study documents, such as the study protocol and clinical study report, are not always available. Proposals should be submitted to DataSharing@Ipsen.com and will be assessed by a scientific review board. Data are available beginning 6 months and ending 5 years after publication; after this time, only raw data may be available.
