# Supplementary material for: Quantification of the endogenous growth hormone and prolactin lowering effects of a somatostatin-dopamine chimera using population PK/PD modeling
Source: J Pharmacokinet Pharmacodyn. 2020 Apr 4;47(3):229–39. doi: 10.1007/s10928-020-09683-3 (PMC7289785; doi:10.1007/s10928-020-09683-3)
Supplement: Supplementary file 1 — Supplementary file1 (DOCX 119 kb) [file 10928_2020_9683_MOESM1_ESM.docx]

Quantification of the endogenous growth hormone and prolactin lowering effects of a somatostatin-dopamine chimera using population PK/PD modeling

Michiel J. van Esdonk, Jacobus Burggraaf, Marion Dehez, Piet H. van der Graaf, Jasper Stevens

*Journal of Pharmacokinetics and Pharmacodynamics*

M.J. van Esdonk; [mvesdonk@chdr.nl](mailto:mvesdonk@chdr.nl); +31 071 524 6400

**Online resource 1 – Pulse frequency stratified per cohort**


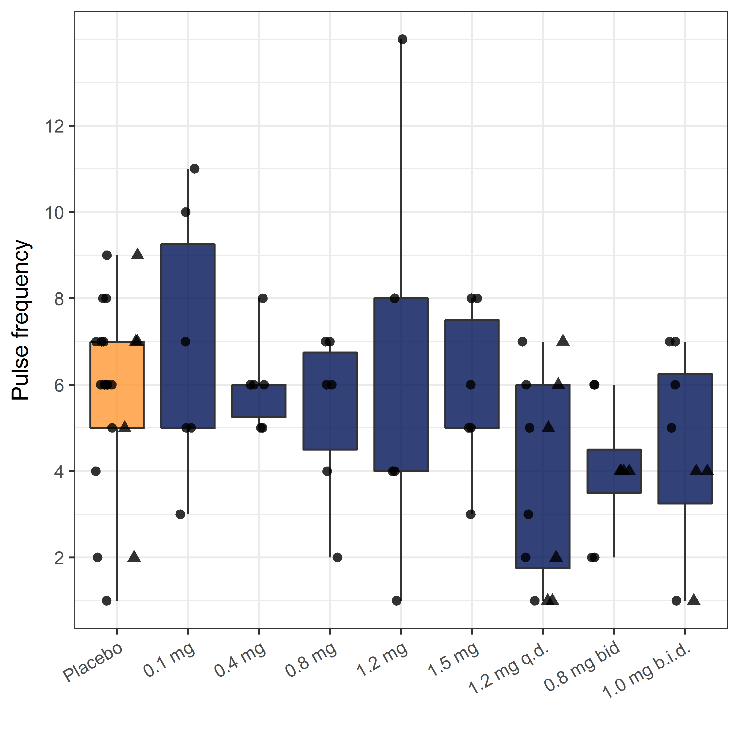


**Number of pulses per cohort. Subjects from multiple ascending dose cohorts were studied on 2 occasions as indicated by the circles (first occasion) and triangles (second occasion). Boxes indicate the 25-75% distribution of the data. Whiskers extend to the minimal and maximal value. Orange color shows the placebo cohort. Blue color shows the BIM23B065 treated cohorts.**
