# Supplementary material for: Quantification of the endogenous growth hormone and prolactin lowering effects of a somatostatin-dopamine chimera using population PK/PD modeling
Source: J Pharmacokinet Pharmacodyn. 2020 Apr 4;47(3):229–39. doi: 10.1007/s10928-020-09683-3 (PMC7289785; doi:10.1007/s10928-020-09683-3)
Supplement: Supplementary file 2 — Supplementary file2 (DOCX 739 kb) [file 10928_2020_9683_MOESM2_ESM.docx]

Quantification of the endogenous growth hormone and prolactin lowering effects of a somatostatin-dopamine chimera using population PK/PD modeling

Michiel J. van Esdonk, Jacobus Burggraaf, Marion Dehez, Piet H. van der Graaf, Jasper Stevens

*Journal of Pharmacokinetics and Pharmacodynamics*

M.J. van Esdonk; [mvesdonk@chdr.nl](mailto:mvesdonk@chdr.nl); +31 071 524 6400

**Online resource 2 – NPDE growth hormone model**

**Model evaluation GH NPDE S.A.D.**

**
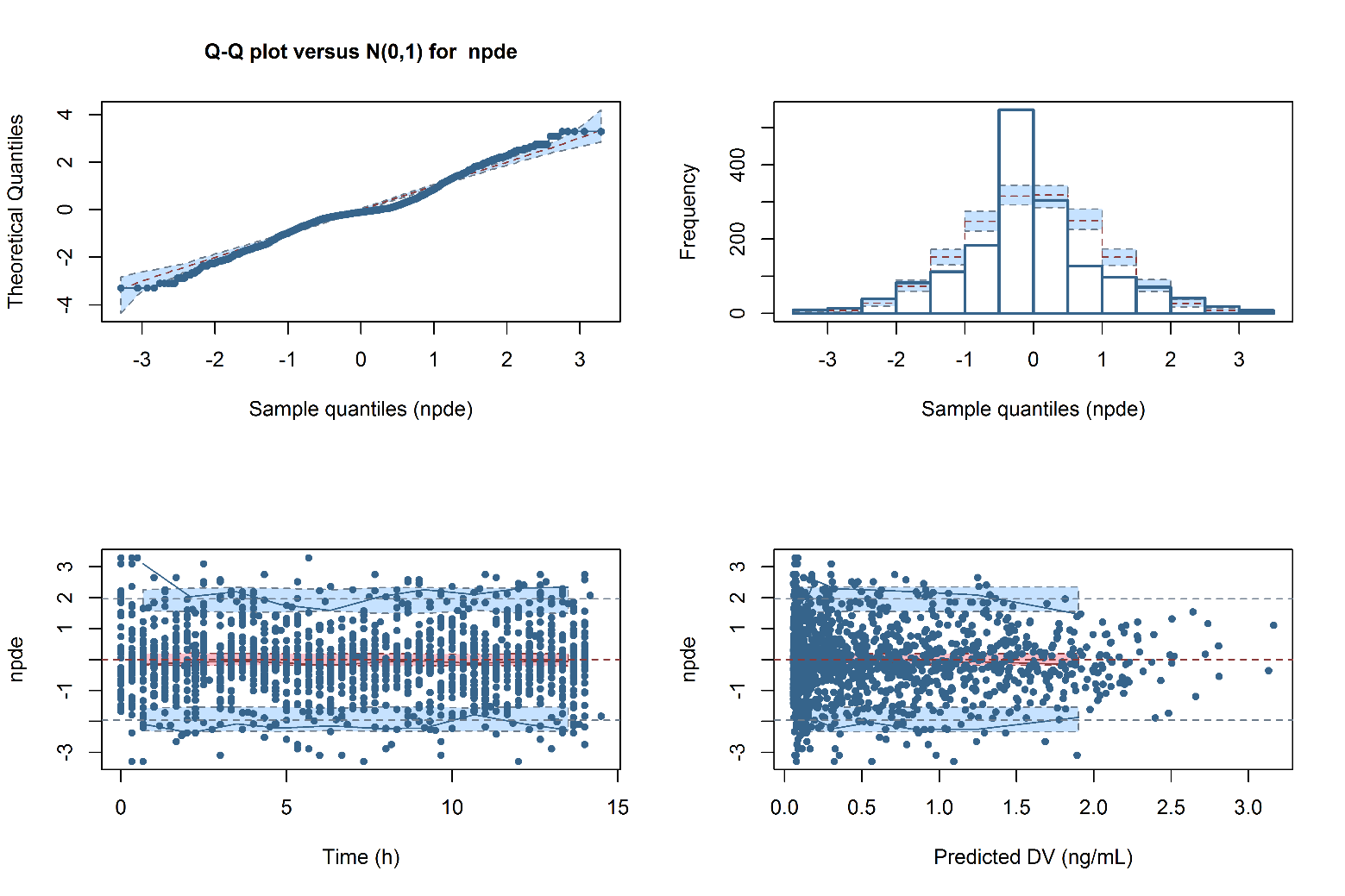
**

**Normalised prediction distribution errors of growth hormone – single ascending dose data. Top left - Quantile-quantile plot of normalised prediction distribution errors, top right - histogram of the normalised prediction distribution errors, bottom left - normalised prediction distribution errors versus time (h), bottom right normalised prediction distribution errors versus population predicted DV.**

**Model evaluation GH NPDE Day 7**

**
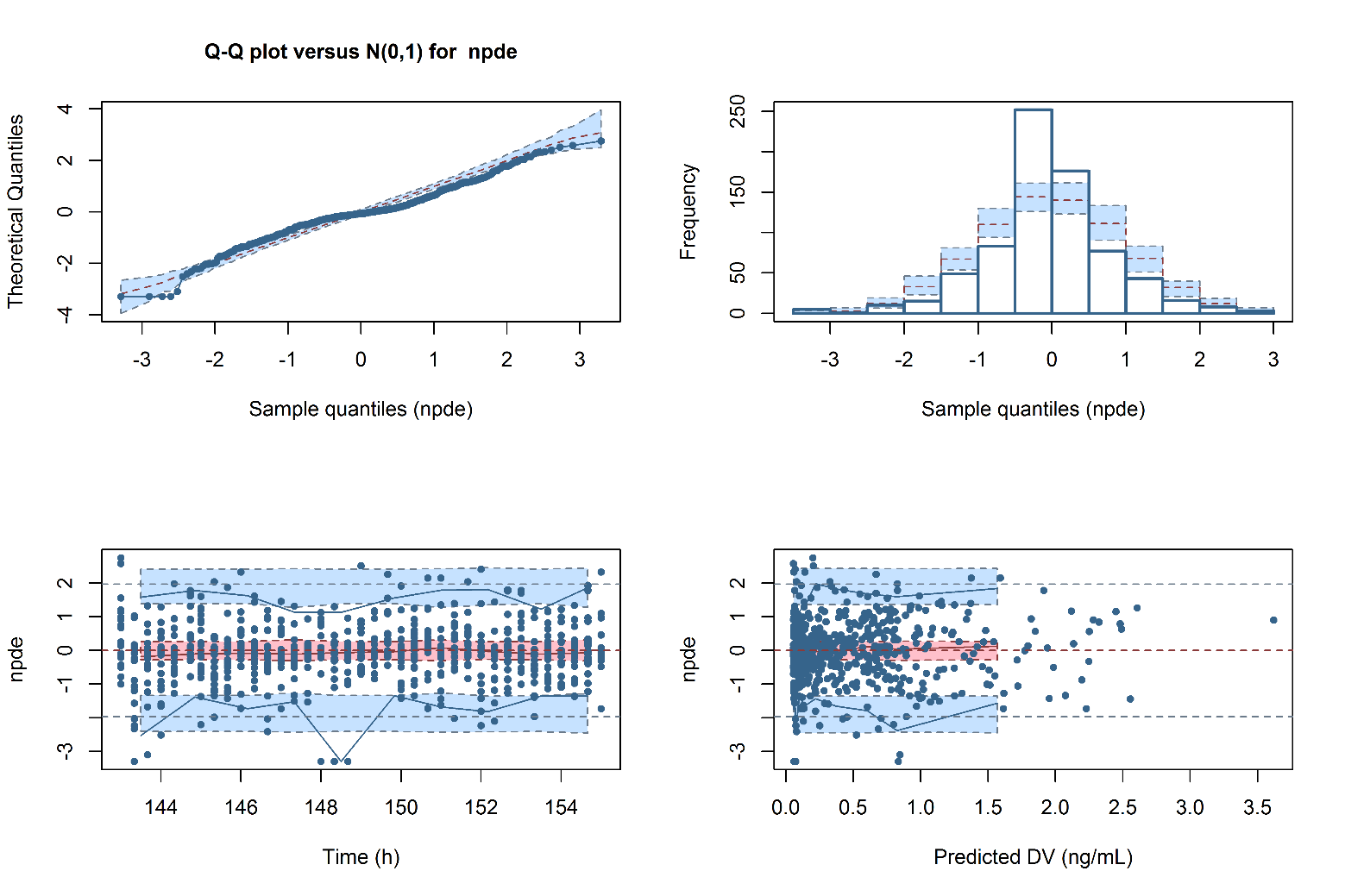
**

**Normalised prediction distribution errors of growth hormone – multiple ascending dose day 7 data. Top left - Quantile-quantile plot of normalised prediction distribution errors, top right - histogram of the normalised prediction distribution errors, bottom left - normalised prediction distribution errors versus time (h), bottom right normalised prediction distribution errors versus population predicted DV.**

**Model evaluation GH NPDE Day 12**

**
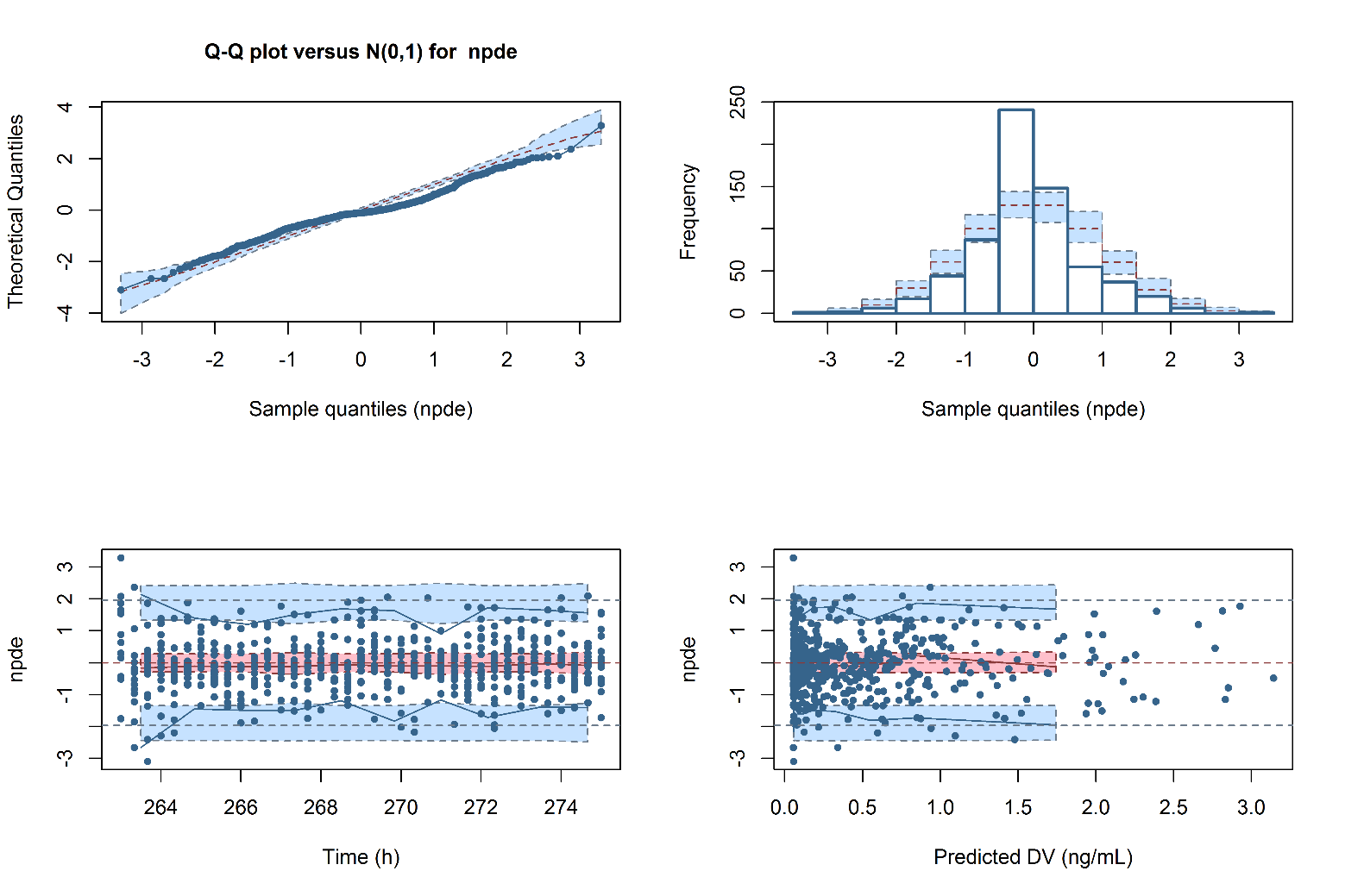
**

**Normalised prediction distribution errors of growth hormone – multiple ascending dose day 12 data. Top left - Quantile-quantile plot of normalised prediction distribution errors, top right - histogram of the normalised prediction distribution errors, bottom left - normalised prediction distribution errors versus time (h), bottom right normalised prediction distribution errors versus population predicted DV.**
