# Supplementary material for: Quantification of the endogenous growth hormone and prolactin lowering effects of a somatostatin-dopamine chimera using population PK/PD modeling
Source: J Pharmacokinet Pharmacodyn. 2020 Apr 4;47(3):229–39. doi: 10.1007/s10928-020-09683-3 (PMC7289785; doi:10.1007/s10928-020-09683-3)
Supplement: Supplementary file 3 — Supplementary file3 (DOCX 288 kb) [file 10928_2020_9683_MOESM3_ESM.docx]

Quantification of the endogenous growth hormone and prolactin lowering effects of a somatostatin-dopamine chimera using population PK/PD modeling

Michiel J. van Esdonk, Jacobus Burggraaf, Marion Dehez, Piet H. van der Graaf, Jasper Stevens

*Journal of Pharmacokinetics and Pharmacodynamics*

M.J. van Esdonk; [mvesdonk@chdr.nl](mailto:mvesdonk@chdr.nl); +31 071 524 6400

**Online resource 3 – Circadian variability prolactin**


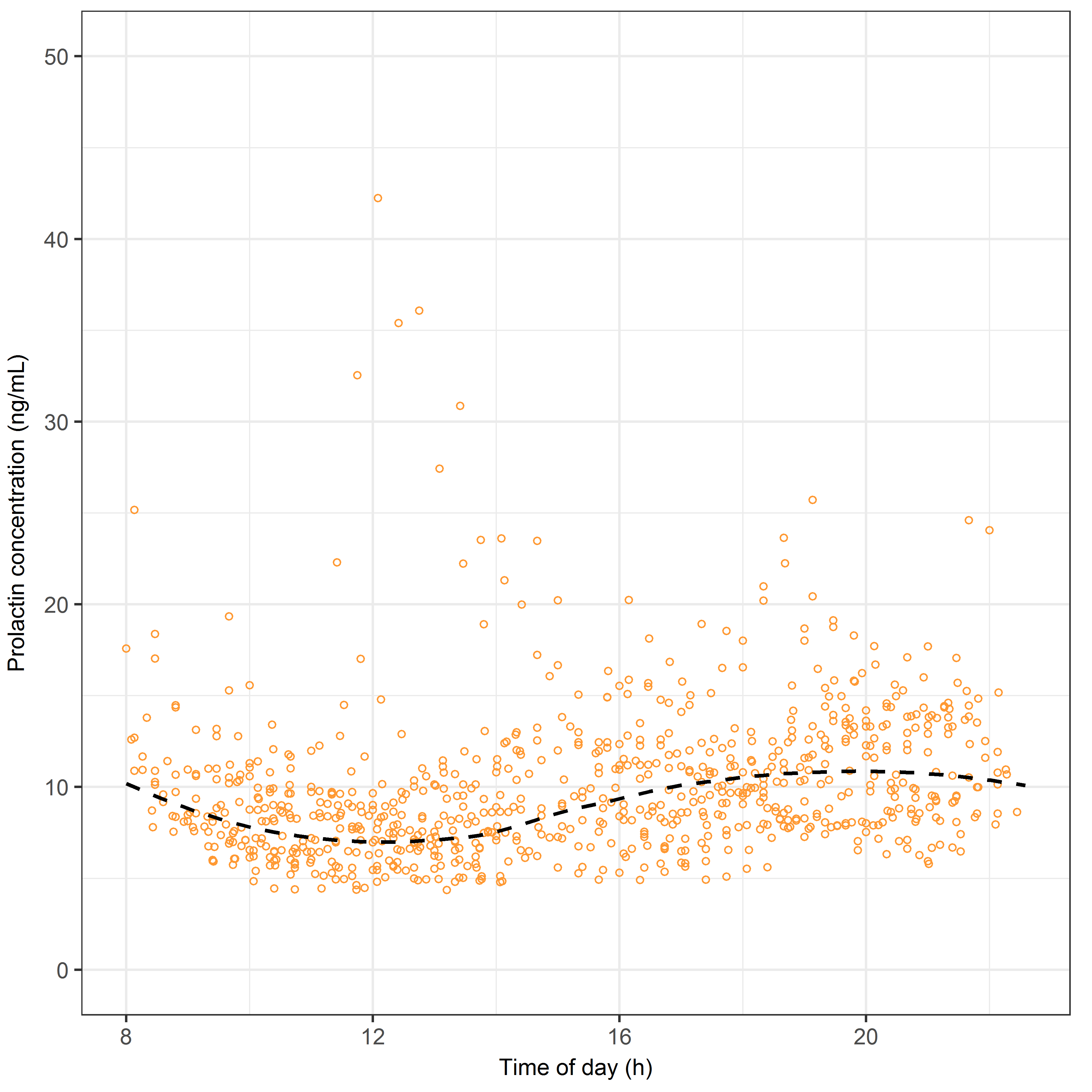


**Exploratory analysis of the prolactin concentrations versus time of day for placebo treated subjects. Dashed black line indicates loess smoother (span = 1).**
