# Supplementary material for: Quantification of the endogenous growth hormone and prolactin lowering effects of a somatostatin-dopamine chimera using population PK/PD modeling
Source: J Pharmacokinet Pharmacodyn. 2020 Apr 4;47(3):229–39. doi: 10.1007/s10928-020-09683-3 (PMC7289785; doi:10.1007/s10928-020-09683-3)
Supplement: Supplementary file 4 — Supplementary file4 (DOCX 494 kb) [file 10928_2020_9683_MOESM4_ESM.docx]

Quantification of the endogenous growth hormone and prolactin lowering effects of a somatostatin-dopamine chimera using population PK/PD modeling

Michiel J. van Esdonk, Jacobus Burggraaf, Marion Dehez, Piet H. van der Graaf, Jasper Stevens

*Journal of Pharmacokinetics and Pharmacodynamics*

M.J. van Esdonk; [mvesdonk@chdr.nl](mailto:mvesdonk@chdr.nl); +31 071 524 6400

**Online resource 4 – Population predictions after multiple dosing**


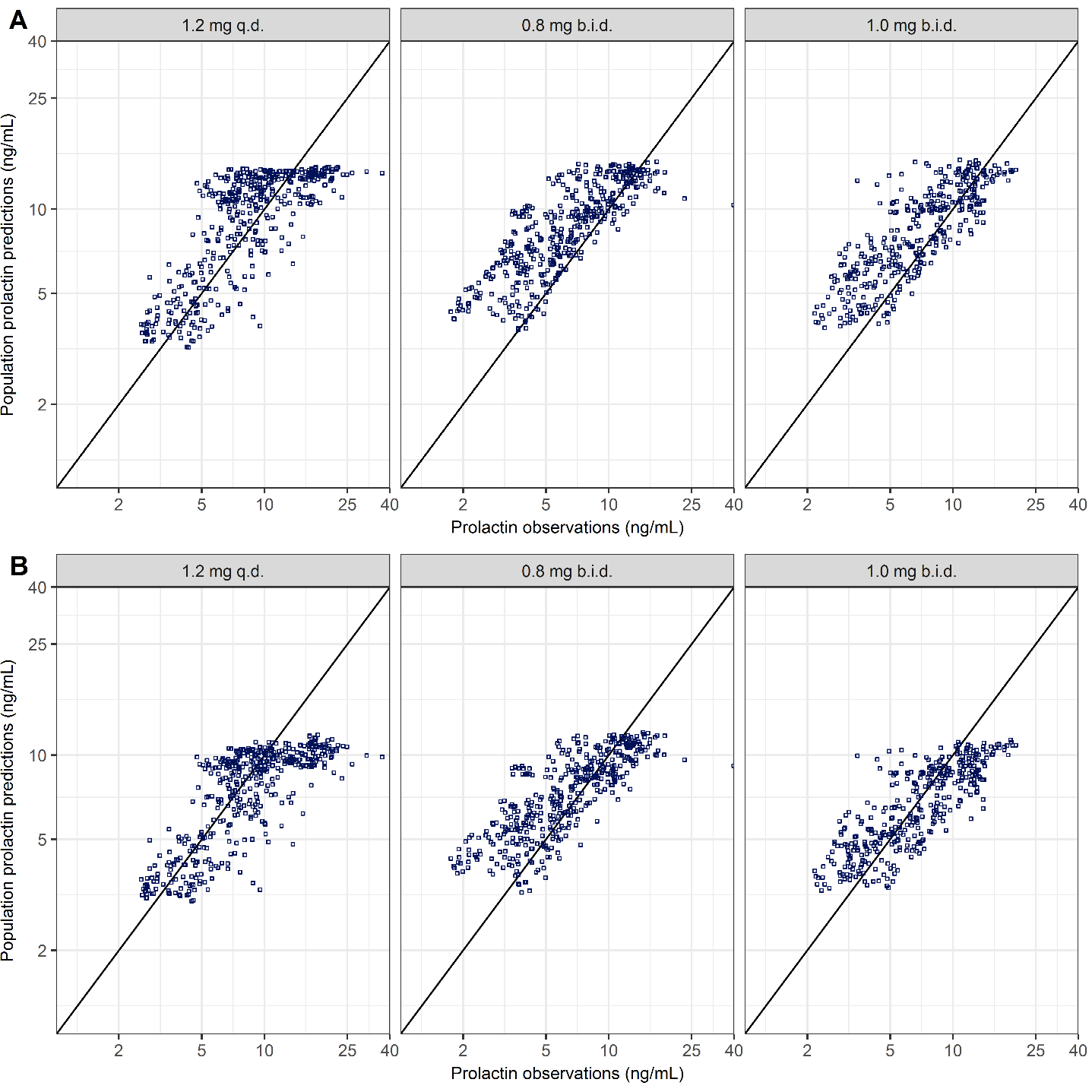


**Population model predictions versus observations for the multiple ascending dose cohorts for the model without the lowering of the k_s-PRL_ over time (A) versus the final model with a correction for the cumulative exposure to BIM23B065 (B). Solid line = line of unity.**
