# Supplementary material for: Quantification of the endogenous growth hormone and prolactin lowering effects of a somatostatin-dopamine chimera using population PK/PD modeling
Source: J Pharmacokinet Pharmacodyn. 2020 Apr 4;47(3):229–39. doi: 10.1007/s10928-020-09683-3 (PMC7289785; doi:10.1007/s10928-020-09683-3)
Supplement: Supplementary file 6 — Supplementary file6 (DOCX 44 kb) [file 10928_2020_9683_MOESM6_ESM.docx]

Quantification of the endogenous growth hormone and prolactin lowering effects of a somatostatin-dopamine chimera using population PK/PD modeling

Michiel J. van Esdonk, Jacobus Burggraaf, Marion Dehez, Piet H. van der Graaf, Jasper Stevens

*Journal of Pharmacokinetics and Pharmacodynamics*

M.J. van Esdonk; [mvesdonk@chdr.nl](mailto:mvesdonk@chdr.nl); +31 071 524 6400

**Online resource 6 – NONMEM model codes**

**Growth hormone model**

$SIZES

LVR=50

$PROBLEM BIM23B065 - PKPD model - 12h GH profiles

$INPUT ID OCC DV MISING DROP CMT MDV AMT EVID TIME DAY FREQ Loc1 Loc2 Loc3 Loc4 Loc5 Loc6 Loc7 Loc8 Loc9 Loc10 Loc11 Loc12 Loc13 Loc14 IVMAX IKM IV2 IQ3 IV3 IKA ICL ICLM IV4 IKT IKT2 IV6 IQ6 WGT HGT BMI AGE SEX MADFLAG1 MADFLAG2 COH PART

$DATA .csv IGNORE=I

$SUBROUTINES ADVAN13 TOL=6

$MODEL

NCOMP=9

COMP=(SCDOSE1) ;1 DOSING CMT

COMP=(CENTRAL) ;2 CENTRAL PLASMA PARENT

COMP=(PERIPH1) ;3 PERIPHERAL PARENT

COMP=(METABOL) ;4 CENTRAL PLASMA METABOLITE

COMP=(TRANSIT) ;5 TRANSIT 1

COMP=(PERIPHM) ;6 PERIPHERAL METABOLITE

COMP=(TRANSIT2) ;7 TRANSIT 2

COMP=(GH) ;8 GH OBSERVATION

COMP=(EFFEC) ;9 Effect CMT

$PK

;PK BIM23B065 (parent) and BIM23B133 (metabolite)

VMAX= IVMAX

KM= IKM

V2= IV2

Q3= IQ3

V3= IV3

KA= IKA

CL= ICL

CLM = ICLM

V4 = IV4

Q6 = IQ6

V6 = IV6

KT = IKT

KT2 = IKT2

; Rate constants

K23=Q3/V2

K32=Q3/V3

K20=CL/V2

K40=CLM/V4

K46=Q6/V4

K64=Q6/V6

; Define between occasion variability

IF(OCC.EQ.1) THEN

BOV1 = ETA(31)

BOV2 = ETA(33)

BOV3 = ETA(35)

ENDIF

IF(OCC.EQ.2) THEN

BOV1 = ETA(32)

BOV2 = ETA(34)

BOV3 = ETA(36)

ENDIF

; PD

TVBASELINE = THETA(1)

BASELINE =TVBASELINE*EXP(ETA(1)+BOV1) ;baseline

TVkout = THETA(2)

kout = TVkout*EXP(ETA(2)+BOV2) ;kout

TVSW = THETA(3)

SW = TVSW*EXP(ETA(3)+BOV3)

TVAMP = THETA(4)

IAMP = TVAMP*EXP(ETA(4))

kin = BASELINE*kout

; Only in part 1 estimate the initial amount

IF(PART.EQ.1) THEN

initA = THETA(5)*EXP(ETA(5))

ENDIF

IF(PART.EQ.2) THEN

initA = 0

ENDIF

;GH compartment initialize on baseline

A_0(8) = BASELINE+initA

;Scaling factor for GH compartment (no volume of distribution available)

S8=1

; Incorporate and initialize pulses

AMPL1 = 0.0000001

AMPL2 = 0.0000001

AMPL3 = 0.0000001

AMPL4 = 0.0000001

AMPL5 = 0.0000001

AMPL6 = 0.0000001

AMPL7 = 0.0000001

AMPL8 = 0.0000001

AMPL9 = 0.0000001

AMPL10 = 0.0000001

AMPL11 = 0.0000001

AMPL12 = 0.0000001

AMPL13 = 0.0000001

AMPL14 = 0.0000001

AMPLMAD1 = 0.0000001

AMPLMAD2 = 0.0000001

; Occasion 1 has a maximum of 14 peaks

IF(OCC.EQ.1) THEN

ETAPULSE1 = ETA(6)

ETAPULSE2 = ETA(7)

ETAPULSE3 = ETA(8)

ETAPULSE4 = ETA(9)

ETAPULSE5 = ETA(10)

ETAPULSE6 = ETA(11)

ETAPULSE7 = ETA(12)

ETAPULSE8 = ETA(13)

ETAPULSE9 = ETA(14)

ETAPULSE10 = ETA(15)

ETAPULSE11 = ETA(16)

ETAPULSE12 = ETA(17)

ETAPULSE13 = ETA(18)

ETAPULSE14 = ETA(19)

ENDIF

; Occasion 2 has a maximum of 9 peaks

IF(OCC.EQ.2) THEN

ETAPULSE1 = ETA(20)

ETAPULSE2 = ETA(21)

ETAPULSE3 = ETA(22)

ETAPULSE4 = ETA(23)

ETAPULSE5 = ETA(24)

ETAPULSE6 = ETA(25)

ETAPULSE7 = ETA(26)

ETAPULSE8 = ETA(27)

ETAPULSE9 = ETA(28)

ENDIF

; A maximum of 14 pulse formulas should be available

IF(FREQ.GE.1) AMPL1 = IAMP*EXP(ETAPULSE1)

IF(FREQ.GE.2) AMPL2 = IAMP*EXP(ETAPULSE2)

IF(FREQ.GE.3) AMPL3 = IAMP*EXP(ETAPULSE3)

IF(FREQ.GE.4) AMPL4 = IAMP*EXP(ETAPULSE4)

IF(FREQ.GE.5) AMPL5 = IAMP*EXP(ETAPULSE5)

IF(FREQ.GE.6) AMPL6 = IAMP*EXP(ETAPULSE6)

IF(FREQ.GE.7) AMPL7 = IAMP*EXP(ETAPULSE7)

IF(FREQ.GE.8) AMPL8 = IAMP*EXP(ETAPULSE8)

IF(FREQ.GE.9) AMPL9 = IAMP*EXP(ETAPULSE9)

IF(FREQ.GE.10) AMPL10 = IAMP*EXP(ETAPULSE10)

IF(FREQ.GE.11) AMPL11 = IAMP*EXP(ETAPULSE11)

IF(FREQ.GE.12) AMPL12 = IAMP*EXP(ETAPULSE12)

IF(FREQ.GE.13) AMPL13 = IAMP*EXP(ETAPULSE13)

IF(FREQ.GE.14) AMPL14 = IAMP*EXP(ETAPULSE14)

; Add 1 pulses for ID that have a peak before the start of observations at Day 7

IF(MADFLAG1.EQ.1) THEN

ETAMAD1 = ETA(29)

AMPLMAD1 = IAMP*EXP(ETAMAD1)

LocMAD1 = THETA(6)

ENDIF

IF(MADFLAG2.EQ.1) THEN

ETAMAD2 = ETA(30)

AMPLMAD2 = IAMP*EXP(ETAMAD2)

LocMAD2 = THETA(7)

ENDIF

;; Drug effect

EMAX = THETA(8)*EXP(ETA(37))

EC50 = THETA(9)*EXP(ETA(38))

GAMMA = THETA(10)*EXP(ETA(39))

; Effect rate constant

KE0 = THETA(11)*EXP(ETA(40))

; Differential equations

$DES

RIN1 = 0

RIN2 = 0

RIN3 = 0

RIN4 = 0

RIN5 = 0

RIN6 = 0

RIN7 = 0

RIN8 = 0

RIN9 = 0

RIN10 = 0

RIN11 = 0

RIN12 = 0

RIN13 = 0

RIN14 = 0

RINMAD1 = 0

RINMAD2 = 0

C=A(2)/V2

CP=A(4)/V4

EFF = 0

IF(A(9).GT.0) EFF = (EMAX*(A(9)**GAMMA)) / (EC50**GAMMA + A(9)**GAMMA)

IF(FREQ.GE.1) RIN1 = 0+EXP(LOG(AMPL1)-0.5*((T-Loc1)/SW)**2)*(1-EFF) ; Surge function with drug effect

IF(FREQ.GE.2) RIN2 = 0+EXP(LOG(AMPL2)-0.5*((T-Loc2)/SW)**2)*(1-EFF)

IF(FREQ.GE.3) RIN3 = 0+EXP(LOG(AMPL3)-0.5*((T-Loc3)/SW)**2)*(1-EFF)

IF(FREQ.GE.4) RIN4 = 0+EXP(LOG(AMPL4)-0.5*((T-Loc4)/SW)**2)*(1-EFF)

IF(FREQ.GE.5) RIN5 = 0+EXP(LOG(AMPL5)-0.5*((T-Loc5)/SW)**2)*(1-EFF)

IF(FREQ.GE.6) RIN6 = 0+EXP(LOG(AMPL6)-0.5*((T-Loc6)/SW)**2)*(1-EFF)

IF(FREQ.GE.7) RIN7 = 0+EXP(LOG(AMPL7)-0.5*((T-Loc7)/SW)**2)*(1-EFF)

IF(FREQ.GE.8) RIN8 = 0+EXP(LOG(AMPL8)-0.5*((T-Loc8)/SW)**2)*(1-EFF)

IF(FREQ.GE.9) RIN9 = 0+EXP(LOG(AMPL9)-0.5*((T-Loc9)/SW)**2)*(1-EFF)

IF(FREQ.GE.10) RIN10 = 0+EXP(LOG(AMPL10)-0.5*((T-Loc10)/SW)**2)*(1-EFF)

IF(FREQ.GE.11) RIN11 = 0+EXP(LOG(AMPL11)-0.5*((T-Loc11)/SW)**2)*(1-EFF)

IF(FREQ.GE.12) RIN12 = 0+EXP(LOG(AMPL12)-0.5*((T-Loc12)/SW)**2)*(1-EFF)

IF(FREQ.GE.13) RIN13 = 0+EXP(LOG(AMPL13)-0.5*((T-Loc13)/SW)**2)*(1-EFF)

IF(FREQ.GE.14) RIN14 = 0+EXP(LOG(AMPL14)-0.5*((T-Loc14)/SW)**2)*(1-EFF)

IF(MADFLAG1.EQ.1) RINMAD1 = 0+EXP(LOG(AMPLMAD1)-0.5*((T-LocMAD1)/SW)**2)*(1-EFF)

IF(MADFLAG2.EQ.1) RINMAD1 = 0+EXP(LOG(AMPLMAD2)-0.5*((T-LocMAD2)/SW)**2)*(1-EFF)

SECRETION = RIN1+RIN2+RIN3+RIN4+RIN5+RIN6+RIN7+RIN8+RIN9+RIN10+RIN11+RIN12+RIN13+RIN14+RINMAD1+RINMAD2

; PK

DADT(1)= -KA*A(1)

DADT(2)= KA*A(1) -K23*A(2)+K32*A(3) - VMAX*C/(KM+C) - K20*A(2)

DADT(3)= K23*A(2)-K32*A(3)

DADT(4)= KT*A(5) + KT2*A(7)- K40*A(4)-K46*A(4) + K64*A(6)

DADT(5)= K20*A(2)-KT*A(5)

DADT(6)= K46*A(4) - K64*A(6)

DADT(7)= VMAX*C/(KM+C)- KT2*A(7)

; PD

DADT(8) = kin + SECRETION - kout*A(8)

; Effect CMT

DADT(9) = KE0*A(2)-KE0*A(9)

$ERROR

IPRE=F

Y=IPRE*(1+EPS(1))

CBIM=A(2)/V2

$THETA

0.056 ; 1, baseline

3.6 ; 2, kel-GH

0.184 ; 3, Secretion width (h)

1.69 ; 4, Amplitude

0.0544 ; 5, A(0)

143 FIX ; 6, Location of peak MAD1 (h)

263 FIX ; 7, Location of peak MAD2 (h) ; Not there in placebo

0.648 ; 8, EMAX

0.609 ; 9, EC50

1 FIX ; 10, GAMMA

1.25 ; 11, KE0

$OMEGA

0.0288 ; 1, baseline

0.225 ; 2, kel-GH

0.0434 ; 3, Secretion width (h)

0 FIX ; 4, Amplitude

0.797 ; 5, A(0) initial amount in GH compartment

$OMEGA BLOCK(1) 3.46 FIX ; BOV on all peaks

$OMEGA BLOCK(1) SAME

$OMEGA BLOCK(1) SAME

$OMEGA BLOCK(1) SAME

$OMEGA BLOCK(1) SAME

$OMEGA BLOCK(1) SAME

$OMEGA BLOCK(1) SAME

$OMEGA BLOCK(1) SAME

$OMEGA BLOCK(1) SAME

$OMEGA BLOCK(1) SAME

$OMEGA BLOCK(1) SAME

$OMEGA BLOCK(1) SAME

$OMEGA BLOCK(1) SAME

$OMEGA BLOCK(1) SAME

$OMEGA BLOCK(1) SAME ; OCC 2 pulses

$OMEGA BLOCK(1) SAME

$OMEGA BLOCK(1) SAME

$OMEGA BLOCK(1) SAME

$OMEGA BLOCK(1) SAME

$OMEGA BLOCK(1) SAME

$OMEGA BLOCK(1) SAME

$OMEGA BLOCK(1) SAME

$OMEGA BLOCK(1) SAME

$OMEGA

0.1 ; BOV on peaks occasion 1 - MAD

0.1 ; BOV on peaks occasion 2 - MAD ; Not there in placebo

$OMEGA BLOCK(1) 0 FIX ; BOV on baseline

$OMEGA BLOCK(1) SAME

$OMEGA BLOCK(1) 0.00775 FIX ; BOV on kout

$OMEGA BLOCK(1) SAME

$OMEGA BLOCK(1) 0.104 FIX ; BOV on SW

$OMEGA BLOCK(1) SAME

$OMEGA

0 FIX ; EMAX

0 FIX ; EC50

0 FIX ; GAMMA

0 FIX ; KE0

$SIGMA

0.0247 ; prop

$EST PRINT=5 MAX=9999 METHOD=1 INTERACTION POSTHOC NOABORT MSFO=mfi

$COV PRINT=E

**Prolactin model**

$PROBLEM BIM23B065 PKPD prolactin secretion

$INPUT ID DROP DV PART COH TRT AMT CMT EVID MDV RATE TAFD IVMAX IKM IV2 IQ3 IV3 IKA ICL ICLM IV4 IKT IKT2 IV6 IQ6 WGT HGT BMI AGE SEX LDV OCC TIME

$DATA .csv IGNORE=I

$SUBROUTINES ADVAN9 TOL=6

$MODEL

COMP=(SCDOSE1) ;1 DOSING CMT

COMP=(CENTRAL) ;2 CENTRAL PLASMA PARENT

COMP=(PERIPH1) ;3 PERIPHERAL PARENT

COMP=(METABOL) ;4 CENTRAL PLASMA METABOLITE

COMP=(TRANSIT) ;5 TRANSIT 1

COMP=(PERIPHM) ;6 PERIPHERAL METABOLITE

COMP=(TRANSIT2) ;7 TRANSIT 2

COMP=(PRL) ;8 PROLACTIN OBSERVATION

COMP=(POOL) ;9 POOL CMT

COMP=(TOLER) ;10 TOLERANCE CMT

$PK

;PK BIM23B065 (parent) and BIM23B133 (metabolite)

VMAX= IVMAX

KM= IKM

V2= IV2

Q3= IQ3

V3= IV3

KA= IKA

CL= ICL

CLM = ICLM

V4 = IV4

Q6 = IQ6

V6 = IV6

KT = IKT

KT2 = IKT2

; Rate constants

K23=Q3/V2

K32=Q3/V3

K20=CL/V2

K40=CLM/V4

K46=Q6/V4

K64=Q6/V6

; PD

kbase = THETA(1)*EXP(ETA(1)) ; prolactin release rate

K80 = THETA(2)*EXP(ETA(2)) ; Elimination rate

AMP1 = THETA(3)*EXP(ETA(3)) ; Amplitude cos 24h

PHSH1 = THETA(4)+ETA(4) ; Phase shift cos 24h

AMP2 = THETA(5)*EXP(ETA(5)) ; Amplitude cos 12h

PHSH2 = THETA(6)+ETA(6) ; Phase shift cos 12h

RFORM = THETA(7)*EXP(ETA(7)) ; Synthesis rate prolactin

EMAX = (THETA(8))*EXP(ETA(8)) ; Maximal inhibitory effect

EC50= THETA(9)*EXP(ETA(9)) ; EC50

IF(COH.LT.20) SF = 0 ; Only add tolerance for MAD cohorts

IF(COH.GT.20) SF = THETA(10)*EXP(ETA(10))

;PRL

A_0(8) = RFORM/K80 ; Initialize PRL compartment

;POOL

A_0(9) = RFORM/kbase ; Initialize pool PRL compartment

$DES

; Add diurnal variation equation with 2 cosine functions

DIU = AMP1*COS(2*3.14159*(TIME-PHSH1)/24)+AMP2*COS(2*3.14159*(TIME-PHSH2)/12)

C=A(2)/V2 ; parent concentrations

CP=A(4)/V4 ; metabolite concentrations

IF(A(2).EQ.0) C=0

IF(A(4).EQ.0) CP=0

; PK

DADT(1)= -KA*A(1)

DADT(2)= KA*A(1) -K23*A(2)+K32*A(3) - VMAX*C/(KM+C) - K20*A(2)

DADT(3)= K23*A(2)-K32*A(3)

DADT(4)= KT*A(5) + KT2*A(7)- K40*A(4)-K46*A(4) + K64*A(6)

DADT(5)= K20*A(2)-KT*A(5)

DADT(6)= K46*A(4) - K64*A(6)

DADT(7)= VMAX*C/(KM+C) - KT2*A(7)

; PD

EFF = C*EMAX/(EC50+C) ; Emax relationship

DADT(8) = kbase*(1-EFF)*(1+DIU)*A(9) - A(8)*K80 ; prolactin plasma compartment

DADT(9) = RFORM*(1-A(10)*SF/(1+A(10)*SF)) - kbase*(1-EFF)*(1+DIU)*A(9) ; Pool compartment

; TOLERANCE effect compartment which accumulates total exposure to BIM23B065

DADT(10)= C

$ERROR

IPRE=F

Y=IPRE*(1+EPS(1))

$THETA

0.011 ; 1, PRL release rate

1.25 ; 2, K80 Elimination rate PRL

0.168 ; 3, AMP1

17.3 ; 4, PHSH1

0.095 ; 5, AMP2

10.2 ; 6, PHSH2

13.3 ; 7, pool formation rate

0.91 ; 8, EMAX

1.27 ; 9, EC50

2.73 ; 10, SF

$OMEGA

0 FIX

0 FIX

0.566

0 FIX

0.868

0 FIX

0.0675

0 FIX

0 FIX

0 FIX

$SIGMA

0.0493 ; Proportional residual error

$EST PRINT=5 MAX=9999 METHOD=1 INTERACTION POSTHOC NOABORT MSFO=mfi

$COV PRINT=E
